# Similarity and consistency assessment of three major online drug–drug interaction resources

**DOI:** 10.1111/bcp.15341

**Published:** 2022-04-12

**Authors:** Elpida Kontsioti, Simon Maskell, Amina Bensalem, Bhaskar Dutta, Munir Pirmohamed

**Affiliations:** ^1^ Department of Electrical Engineering and Electronics University of Liverpool Liverpool UK; ^2^ Institute for Risk and Uncertainty University of Liverpool Liverpool UK; ^3^ R&D, Ceva Santé Animale Libourne France; ^4^ Patient Safety Center of Excellence AstraZeneca Gaithersburg MD USA; ^5^ The Wolfson Centre for Personalized Medicine, MRC Centre for Drug Safety Science, Department of Pharmacology and Therapeutics, Institute of Systems, Molecular and Integrative Biology University of Liverpool Liverpool UK

**Keywords:** clinical decision support, clinical management of drug interactions, drug information, drug–drug interaction, drug–drug interaction software

## Abstract

**Aims:**

The aim of this study was to explore the level of agreement on drug–drug interaction (DDI) information listed in three major online drug information resources (DIRs) in terms of: (1) interacting drug pairs; (2) severity rating; (3) evidence rating; and (4) clinical management recommendations.

**Methods:**

We extracted information from the *British National Formulary* (BNF), *Thesaurus* and *Micromedex*. Following drug name normalisation, we estimated the overlap of the DIRs in terms of DDI. We annotated clinical management recommendations either manually, where possible, or through application of a machine learning algorithm.

**Results:**

The DIRs contained 51 481 (*BNF*), 38 037 (*Thesaurus*) and 65 446 (*Micromedex*) drug pairs involved in DDIs. The number of common DDIs across the three DIRs was 6970 (13.54% of *BNF*, 18.32% of *Thesaurus* and 10.65% of *Micromedex*). *Micromedex* and *Thesaurus* overall showed higher levels of similarity in their severity ratings, while the *BNF* agreed more with *Micromedex* on the critical severity ratings and with *Thesaurus* on the least significant ones. Evidence rating agreement between *BNF* and *Micromedex* was generally poor. Variation in clinical management recommendations was also identified, with some categories (i.e., *Monitor* and *Adjust dose*) showing higher levels of agreement compared to others (i.e., *Use with caution*, *Wash‐out*, *Modify administration*).

**Conclusions:**

There is considerable variation in the DDIs included in the examined DIRs, together with variability in categorisation of severity and clinical advice given. DDIs labelled as critical were more likely to appear in multiple DIRs. Such variability in information could have deleterious consequences for patient safety, and there is a need for harmonisation and standardisation.

**What is already known about this subject**

- There is a variety of online DIRs, which differ in coverage, content and inclusion criteria, that are available to clinicians and other prescribers, mainly for prescribing decision support purposes.
- Previous studies have described major discrepancies between widely used DIRs on inclusion of critical DDIs or interactions of specific therapeutic categories, along with discordance in their severity and evidence ratings.

**What this study adds**

- To the best of our knowledge, this is the first study to concurrently compare the similarity among complete datasets from DIRs in terms of inclusion of drug pairs, recommendations for clinical management, severity and evidence of DDIs.
- Considerable variation was identified in all types of information for DDIs, which has important clinical implications for patient safety and requires efforts towards harmonisation and standardisation.

## 1 INTRODUCTION

Coadministration of multiple drugs increases the risk of drug–drug interactions (DDIs). A DDI can be defined as the modification in the therapeutic effect of one or more medications due to the presence of concomitant medications, and can lead to clinically significant events, caused by either an increase in the effect of the interacting drug leading to an adverse drug reaction (ADR), or a decrease in its effect that results in lack of efficacy. Previous studies have reported that DDIs are a significant cause of hospitalisation, being responsible for 16.6% of cases where the cause was an ADR and around 1% of all hospital admissions.^1^ The risk for DDIs increases during hospitalisation and after discharge, as there is a high prevalence of administration of potentially interacting drug combinations.^2^

As new medicines gain approval each year, the volume of possible drug combinations is constantly growing. At the same time, the rising numbers of people with multimorbidity together with increasing life expectancy around the world are associated with the phenomenon of polypharmacy, which aggravates the impact of DDIs in clinical practice. According to a recent review, more than one in three people in England aged 60 and older are exposed to at least five medicines at the same time, with more than a third of all people above 80 being on eight or more medicines.^3^

The clinical manifestation of DDIs depends on several factors. Potential DDIs, based on pharmacological knowledge, far outnumber those which lead to clinically significant adverse effects.^4^ Despite the theoretical potential for an ADR to occur due to a DDI, there are several factors that can affect the actual behaviour of drug molecules inside the human body, including dosage and patient characteristics (e.g., age, number and type of morbidities, etc). Also, genetic polymorphisms of drug‐metabolising enzymes, drug transporters or drug receptors may be responsible for the appearance of some DDIs.^5^ Therefore, it is difficult to accurately predict the occurrence of a clinically significant DDI in an individual patient. To overcome this problem, clinicians are commonly aided by drug information resources (DIRs) to assess the risk–benefit ratio of each drug added to the treatment schedule. DIRs can be either open source or commercial, and they are often incorporated in computerised clinical decision support (CDS) tools.

The availability of DIR information related to severity, evidence availability and clinical options for management of DDIs (e.g., entirely avoid the combination, monitor, adjust dose, etc.) are central to the development of CDS.^6^ Inconsistencies between DIRs may confuse clinicians and impact clinical decisions.^7^ Previous studies have assessed the level of agreement of DIRs, mainly in terms of listing of DDIs and severity ratings. However, most of them were restricted to only DDI listing for a limited number of drugs, specific therapeutic categories, or did not focus solely on clinical resources.^8^, ^9^, ^10^ Moreover, the ability of a DIR to identify clinically relevant DDIs or capture critical DDIs (e.g., FDA black box warnings, ONC [Office of the National Coordinator for Health Information Technology] high priority list^11^) has also been explored.^12^, ^13^, ^14^ However, it remains unclear to what extent DIRs from different geographic locations agree on their DDI listings as well as DDI‐related information.

The aim of this study was to assess the concordance of leading clinical resources for DDIs from three different countries of origin in terms of: (1) inclusion of interacting drug pairs; (2) severity rating; (3) evidence rating; and (4) clinical management recommendations. To the best of our knowledge, this is the first comprehensive study that attempts to compare multiple types of information pertinent to DDIs at the same time across entire DIRs. To ensure clinical utility, only clinically relevant resources were included in the present study (see Figure S1 in the Supporting Information for an overview of online DDI resources). Data sources of potential DDIs (e.g., DrugBank) that are mainly used for scientific research purposes were not taken into consideration.

## 2 METHODS

### 2.1 Data sources

DDI data from two open‐source DIRs and one commercial online DIR were included in our evaluation: the British National Formulary^15^ (hereafter called *BNF*), Interactions Thesaurus^16^ by the French Medicines Agency (Agence Nationale de Sécurité du Médicament et des produits de santé, ANSM) (hereafter called *Thesaurus*) and IBM Micromedex^17^ (hereafter called *Micromedex*). The *BNF* is extensively used in the UK.^18^, ^19^ *Thesaurus* is maintained and updated annually by ANSM, being considered as the official source of information relevant to DDIs for French clinicians. *Micromedex* is a leading clinical information resource, listed as one of the statutorily named compendia in the Medicaid program and is widely used in the United States.^20^, ^21^ The *BNF* and *Thesaurus* are publicly available online, while *Micromedex* can only be accessed via subscription.

### 2.2 Data extraction

Automated web data collection (web scraping) was executed for *BNF* and *Micromedex* in Python 3.6^22^ with terms of use that permit data collection. *Thesaurus* is a portable document format (PDF) file that is curated and updated annually. An R package ( *IMthesaurusANSM* ^23^) enabled the automatic data extraction from the original document (version September 2019). The types of extracted information from each DIR are summarised in Table 1.

**TABLE 1:** Extracted information from the drug information resources

DIR	Extracted fields	Categories
BNF (accessed June 2018)	Drug name Interactant name Description Severity (where present) Evidence (where present)	(a) Active pharmaceutical ingredients (APIs) (e.g., atropine); (b) Drug classes (e.g., combined hormonal contraceptives); (c) Herbs and supplements (e.g., peppermint oil); (d) Foods and beverages (e.g., grapefruit juice).
Thesaurus ‐ September 2019 update (accessed July 2020)	Drug name Interactant name Mechanism (if available) Severity index Additional information (specification for drug class, etc.) Clinical information (i.e., manifestation, management)	(a) Drug ingredient; (b) Drug classes.
Micromedex In‐depth answers database (detailed evidence‐based information) (accessed August 2018)	Drug name Interactant name Interaction effect Summary Severity Onset Substantiation Clinical management	(a) Drug ingredients; (b) Combination drugs; (c) Food; (d) Tobacco; (e) Lab tests.

We mapped DDIs from *Thesaurus* at the drug class level (e.g., beta blockers) to their constituent individual drug ingredients using a mapping table available on the ANSM website. We also excluded DDIs from *Micromedex* containing drug combinations (e.g., hydroxyamphetamine/tropicamide), as those simply collated DDIs from the combination's individual ingredients; hence, only single ingredient drug interactions were considered. Also, cases where drug names of an interacting pair were swapped (i.e. [*D*1, *D*2] and [*D*2, *D*1]) were considered equivalent and duplicate entries were removed from the tables that stored the extracted data (*BNF original table*, *Thesaurus original table* and *Micromedex original table*).

### 2.3 Drug name normalisation

Initial drug names were normalised to RxNorm Ingredients (for US‐marketed medicines)^24^ and RxNorm Extension Ingredients (for medicines not found in RxNorm)^25^ using the Observational Health Data Sciences and Informatics (OHDSI) Usagi tool.^26^ Some names were too general to be mapped (e.g., *insulins*), or were not present in either vocabulary. Thus, interacting pairs containing at least one unmapped drug were excluded from the corresponding DIR table. As the scope of this study was limited to DDIs, only interacting pairs containing drugs were included in the final DIR tables and interactions with herbs, alcohol, food, etc., were excluded. The final tables (*BNF final table*, *Thesaurus final table* and *Micromedex final table*) contained drug interacting pairs and associated information based on normalised drug names. Any duplicate entries based on common normalised names were combined into a single entry. For example, *Metoprolol Tartrate* and *Metoprolol Succinate* were both mapped to the RxNorm entity *Metoprolol*, and their interactions were merged to produce a single set.

### 2.4 Comparison of resources

#### 2.4.1 Listing of DDIs

The pairwise and three‐way overlaps of the final DIR tables were estimated by calculating counts of common drug pairs across the DIRs as well as coverage rates (i.e., the percentage of a set A covered by B, where B is a subset of A). The directionality of interacting drug pairs was not taken into account (i.e., [*D*1, *D*2] and [*D*2, *D*1] were considered equivalent). A *DIR intersection list* containing common interacting drug pairs among all three DIRs with their corresponding text descriptions from each source was generated.

#### 2.4.2 Severity and evidence ratings

All three DIRs included severity ratings (Table 2a), while only the *BNF* and *Micromedex* contained separate text fields regarding evidence ratings (Table 2b). Some DDIs from *Thesaurus* appeared at the drug class level in the original source, which was associated with multiple severity ratings; thus, individual drugs were assigned all applicable ratings from the drug class during the mapping process. Also, some DDIs were linked to multiple severity ratings, based on the clinical circumstances (e.g., route of administration, dose, etc.). In all cases where multiple ratings were available for an individual DDI, the highest one was kept for further analysis.

**TABLE 2:** Information contained in drug information resources on drug–drug interactions relating to (a) the severity ratings (in descending order as displayed in the original source); and (b) the evidence ratings

DIR	Level	Definition
Severity		
BNF	1 – Severe^a^	The result may be a life‐threatening event or have a permanent detrimental effect.
	2 – Moderate	The result could cause considerable distress or partially incapacitate a patient; they are unlikely to be life‐threatening or result in long‐term effects.
	3 – Mild	The result is unlikely to cause concern or incapacitate the majority of patients.
	4 – Unknown	Used for those interactions that are predicted, but there is insufficient evidence to hazard a guess at the outcome.
Thesaurus	1 – Contraindicated^a^	
	2 – Not recommended^a^	
	3 – Precautions for use	
	4 – Take into consideration	
Micromedex	1 – Contraindicated^a^	The drugs are contraindicated for concurrent use.
	2 – Major^a^	The interaction may be life‐threatening and/or require medical intervention to minimise or prevent serious adverse effects.
	3 – Moderate	The interaction may result in exacerbation of the patient's condition and/or require an alteration in therapy.
	4 – Minor	The interaction would have limited clinical effects. Manifestations may include an increase in the frequency or severity of the side effects but generally would not require a major alteration in therapy.
Evidence		
BNF	Study	For interactions where the information is based on formal study including those for other drugs with same mechanism (e.g., known inducers, inhibitors or substrates of cytochrome P450 isoenzymes or P‐glycoprotein).
	Anecdotal	Interactions based on either a single case report or a limited number of case reports.
	Theoretical	Interactions that are predicted based on sound theoretical considerations. The information may have been derived from in vitro studies or based on the way other members in the same class act.
Micromedex	Established (excellent)	Controlled studies have clearly established the existence of the interaction.
	Theoretical (good)	Documentation strongly suggests the interactions exists, but well‐controlled studies are lacking.
	Probable (poor)	Available documentation is poor, but pharmacologic considerations lead clinicians to suspect the interaction exists; or, documentation is good for a pharmacologically similar drug.

^a^ Critical severity ratings.

To explore discrepancies among DIRs related to severity and evidence ratings for DDIs, we calculated the subset size, pairwise coverage rates and Jaccard indices for all possible pairs of DIR ratings.

#### 2.4.3 Clinical management recommendations

We aimed to explore the consistency among the clinical management recommendations provided by the DIRs by analysing text descriptions from the *DIR intersection list*. The *BNF* provided a succinct description for each drug pair containing all types of available DDI information in a text field, while *Thesaurus* and *Micromedex* contained separate text fields (*Conduite à tenir* and *Clinical Management*, respectively) under each drug pair related to clinical management options.

Basic pre‐processing involved text conversion to lowercase, drug name blinding (i.e., replacement of all drug names with a common string), and sentence tokenisation using the Natural Language Toolkit (NLTK) in Python 3.6.^27^

The following advice categories were initially considered:

- Avoid;
- Use with caution;
- Space dosing times;
- Wash‐out;
- Monitor;
- Adjust dose;
- Modify administration;
- Use alternative;
- Discontinue.

Cases that recommended clinicians to refer to literature or other resources, without mentioning any concrete clinical advice, were excluded.

The limited number of unique sentences sourced from *BNF* (*n* = 305) and *Thesaurus* (*n* = 387) following drug name blinding enabled manual sentence labelling, with each sentence being classified into one or multiple advice categories.

To annotate *Clinical Management* text descriptions in *Micromedex* (*n* = 4507), we developed a bespoke text classification process in Python using a methodology that has been widely implemented in similar tasks and provided the desired functionality while keeping the level of complexity low (Figure S2 in the Supporting Information). First, we annotated a subset of randomly selected unique sentences (*n* = 200) by considering the above‐mentioned categories. Then, each labelled sentence was tokenised into its constituent tokens (i.e., words) and stemming (i.e., reducing words to their word roots) was applied. We used *term frequency‐inverse document frequency* (*tf‐idf*) to calculate weights for each word in the annotated sentences. The goal of *tf‐idf* is to reduce the impact of very commonly occurring words in a corpus, assuming that they are less informative. Term frequencies are calculated by counting the relative frequency of each word appearing in each of the annotated sentences. Inverse document frequencies of each word (in its root form) are estimates of the overall presence of the word across all sentences (i.e., how commonly or rarely it appears). The formula for calculation of a word's *tf‐idf* is the following:

(1) $$\mathrm{tf}-{\mathrm{idf}}_{w,s}={\mathrm{tf}}_{w,s}\times \left(\log\frac{N}{{\mathrm{df}}_{w}}+1\right)$$

where ${\mathrm{tf}}_{w,s}$ represents the term frequency of the word, *w*, in the sentence, *s* (i.e., the number of times the word appears in the sentence divided by the total number of words in the sentence); N is the total number of sentences in the corpus; and ${\mathrm{df}}_{w}$ is the “document” frequency of the word, *w* (i.e., the number of sentences that contain the specific word). Weights were applied for sentence encoding to feed classifiers that used a supervised machine learning model called *linear kernel Support Vector Machine* (SVM) for binary text classification (i.e., each sentence was classified as to whether it belongs to each of the advice categories under consideration). We applied class weights to account for the imbalanced training sets (i.e., disproportion between the number of positive and negative instances) and used leave‐one‐out cross validation to evaluate performance of the difference classifiers through receiver operating characteristic (ROC) analysis. By estimating the positive predictive value (PPV) for the different thresholds, we excluded sentence classifiers with a PPV below 80% due to poor performance; the remaining, unannotated sentences from *Micromedex* were automatically labelled by the classifiers using the threshold with maximum sensitivity for PPVs above 80%. A subset (*n* = 100) of the automatically annotated sentences (*validation set*) was also manually annotated to independently estimate the classifiers' performance in the total set of *Micromedex* sentences.

## 3 RESULTS

### 3.1 Comparative assessment in terms of listing

*Micromedex* contained the largest number of DDI drug pairs (*n* = 65 446), as well as normalised ingredients involved in DDIs (*n* = 1967), followed by *BNF* (*n* = 51 481) and *Thesaurus* (*n* = 38 037) that covered 984 and 1001 normalised ingredients, respectively, in their DDI section. The collation of the three final DDI tables included 121 351 DDI drug pairs. The counts of initial drug names, normalised drug ingredients and unique DDIs in each DIR are summarised in Table 3.

**TABLE 3:** Number of initial drug names, normalised ingredients, and drug–drug interaction counts per drug information resource

DIR	Initial drug names	Normalised ingredients	DDI counts
**BNF**	1004	984	51 481
**Thesaurus**	1049	1001	38 037
**Micromedex**	2602	1967	65 446

**FIGURE 1:**
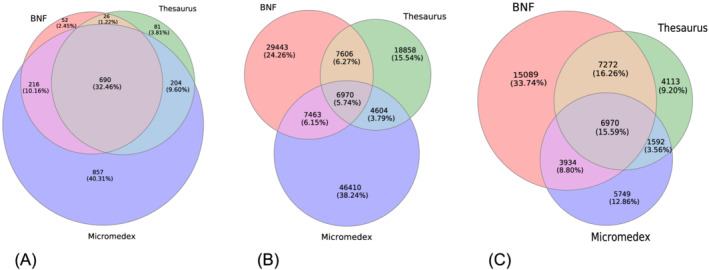
Venn diagrams illustrating the intersections in terms of: (A) drug ingredients; (B) unique drug–drug interaction pairs included in the drug information resources; and (C) drug–drug interaction pairs included in the drug information resources only for the ingredient intersection subset. Each circle represents a drug information resource and their intersections show the number of ingredients/drug–drug interactions they share with each one of the other drug information resources

There were 690 common normalised ingredients involved in DDIs across all examined DIRs, with *BNF* and *Micromedex* sharing the largest number (*n* = 906), followed by *Thesaurus* and *Micromedex* (*n* = 894) and, lastly, the *BNF* and *Thesaurus* (*n* = 716) (Figure 1A). Almost four out of five DDI drug pairs (78.04%, *n* = 94 708) in the collated list were only mentioned by a single DIR, with 57.19% of BNF, 49.58% of *Thesaurus* and 70.91% of *Micromedex* DDI entries missing from the other two DIRs.

The percentage of DDIs mentioned in exactly two out of three DIRs was lower (16.21%, *n* = 19 673). *BNF* shared 14 576 DDIs with *Thesaurus* (28.31% of *BNF*; 38.32% of *Thesaurus*) and 14 433 DDIs with *Micromedex* (28.04% of *BNF*; 22.05% of *Micromedex*), while *Thesaurus* and *Micromedex* had 11 574 common DDIs (30.43% of *Thesaurus*; 17.68% of *Micromedex*). The intersection of the three DIRs in terms of DDIs (*n* = 6970) represented only 5.74% of the collated list, 13.54% of *BNF*, 18.32% of *Thesaurus* and 10.65% of *Micromedex* (Figure 1B).

In terms of DDIs restricted to common ingredients across the three DIRs (*n* = 44 719), more than half (*n* = 24 951) were only found in a single DIR (33.74% in the *BNF* alone, in comparison to 12.86% and 9.20% in *Micromedex* and *Thesaurus*, respectively), while 28.62% were present in two out of three DIRs. In the setting of ingredient‐restricted DDIs, the *BNF* intersected with large proportions of both *Thesaurus* (71.40%) and *Micromedex* (59.76%), while *Thesaurus* overlapped with less than half of *BNF* (42.81%). Finally, the intersection of the three DIRs represented 15.59% of the restricted DDIs (Figure 1C).

### 3.2 Comparative assessment of severity rating

The categorisation of DDIs in each DIR in terms of severity rating is outlined in Table 4. Regarding critical severity rating categories, almost one quarter (24.56%) of unique DDIs in the *BNF* were labelled as *Severe*, compared to 7.75% from *Thesaurus* characterised as *Contraindicated*, and 33.60% as *Not recommended*. In *Micromedex*, 8.76% of unique DDIs were mentioned as *Contraindicated*, while *Major* was the most frequent category (63.73%).

**TABLE 4:** Number and percentage of drug–drug interactions by severity rating in each drug information resource

Severity rating	BNF	Thesaurus	Micromedex
1	12 644 (24.56%)	2949 (7.75%)	5730 (8.76%)
2	4997 (9.46%)	12 779 (33.60%)	41 713 (63.73%)
3	273 (0.51%)	8195 (21.54%)	15 890 (24.28%)
4	33 705 (65.47%)	14 114 (37.11%)	2113 (3.23%)

**FIGURE 2:**
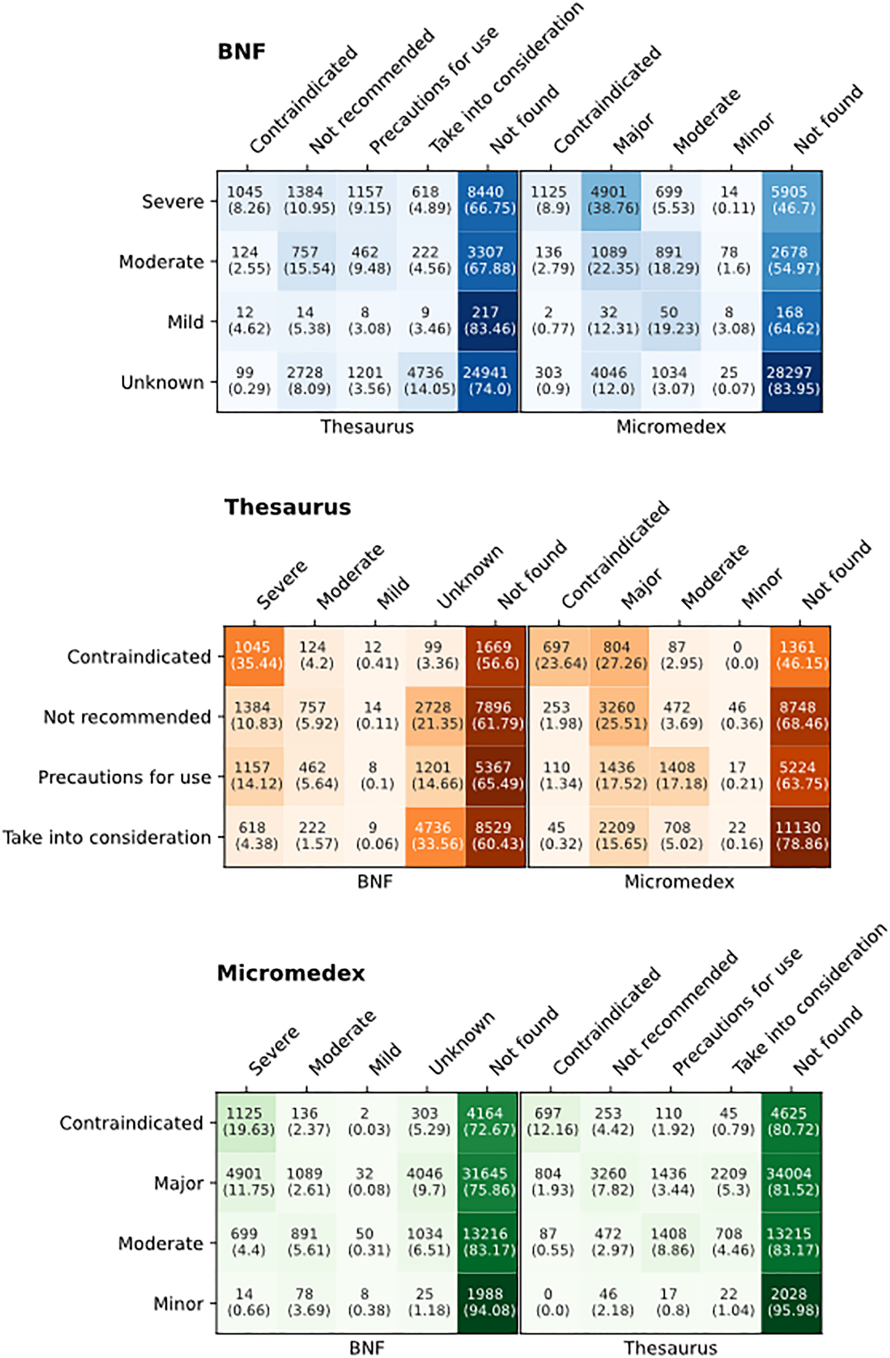
Pairwise comparison tables for the different drug–drug interaction severity levels. For tables (A)–(C), row labels contain the severity ratings of the drug information resource under consideration, while column labels represent the severity ratings of the remaining two drug information resources. A separate column has been added to include the numbers of unique drug–drug interactions missing from each of the other drug information resources. Each row contains the number of unique drug–drug interactions per severity rating of the drug information resource under consideration, subcategorised by the severity ratings of the other drug information resources. The numbers in parentheses represent the corresponding percentages of the various sets per severity rating of the drug information resource under consideration. Colour gradient shows the relative differences in the percentages mentioned among the various overlapping sets

When considering the pairwise DIR overlap using coverage rates (Figure 2), the number of DDIs jointly rated as critical was:

- 2429 between *BNF* and *Thesaurus*, representing 19.21% of the *BNF* and 15.44% of *Thesaurus* critical DDIs;
- 6026 between the *BNF* and *Micromedex* (47.66% of *BNF* and 31.38% of *Micromedex* critical DDIs);
- 5014 between *Thesaurus* and *Micromedex*, covering 78.39% of *Thesaurus* and 26.33% of *Micromedex* critical DDIs);
- 1768 among all three DIRs (25.37% of the *DIR intersection list*).

The percentage of DDIs from the *DIR intersection list* that were considered critical by *BNF*, *Thesaurus* and *Micromedex* was 43.39% (*n* = 3024), 52.32% (*n* = 3647) and 81.51% (*n* = 5681), respectively.

A similarity matrix of the Jaccard index for all DIR severity rating combinations is included in Figure S3 in the Supporting Information.

### 3.3 Comparative assessment of evidence rating

The *BNF* included evidence ratings in just around one third (30.66%) of its DDIs, with the majority being flagged as *Theoretical* (16.99%), followed by *Study* (11.89%) and *Anecdotal* (1.78%). In *Micromedex*, evidence ratings were consistently present under each DDI description. *Theoretical* was the most common category (70.91%), while *Probable* and *Established* included 20.36% and 8.73% of the DDIs mentioned in *Micromedex*.

Almost half (48.13%) of the DDIs from the *DIR intersection list* contained no evidence rating in the *BNF*; the remainder belonged to *Study* (28.05%), *Theoretical* (19.94%) and, lastly, *Anecdotal* (3.87%) evidence categories. According to *Micromedex*, most of them (64.51%) were *Theoretical*, with 19.94% and 15.55% being considered as *Probable* and *Established*, respectively.

**FIGURE 3:**
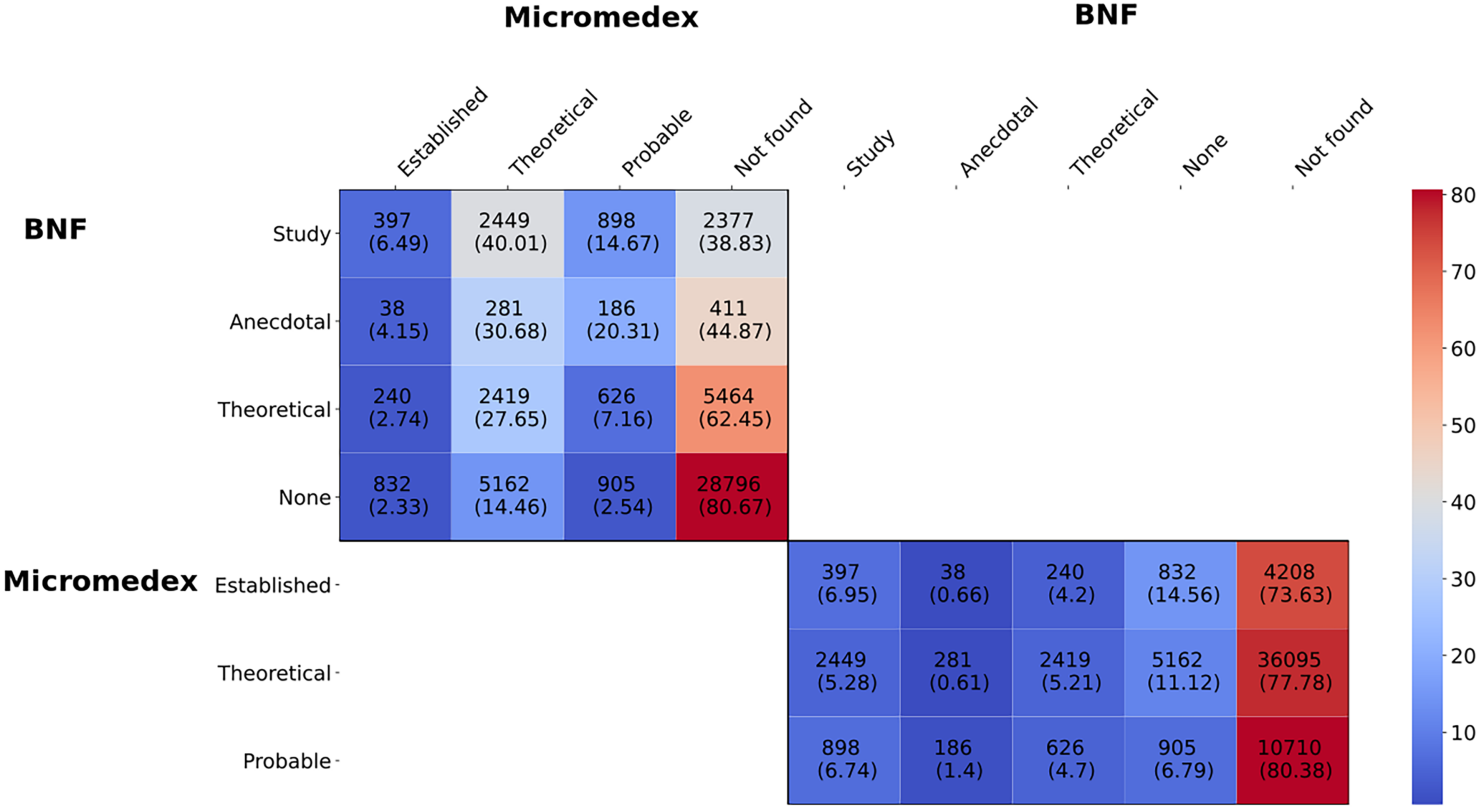
Heatmap for evidence rating comparison between BNF and Micromedex, including counts and coverage rates

Figure 3 shows the overlap of the different evidence categories between the two DIRs as a two‐by‐two grid with subset counts and coverage rates. Probable DDIs from *Micromedex* and DDIs with no evidence rating from *BNF* were absent in higher percentages in the other resource. In both DIRs, the percentage of missing DDIs increased as one moved towards DDIs with a “poorer” or no evidence rating in the other resource. Using the Jaccard index, agreement between ratings was generally low in all cases, with the *BNF Study* and *Micromedex Theoretical* categories being the most similar (0.04662), while the *BNF Anecdotal* and *Micromedex Established* had the lowest concordance (0.00573).

### 3.4 Comparative assessment of clinical management advice

In the *BNF*, no instances of the *Discontinue* advice category were identified in the *DIR intersection list*, while in *Micromedex*, counts for seven out of the nine advice categories are provided, as no sentence classifier was applied to extrapolate the remaining labels (i.e., *Space dosing times* and *Modify administration*) due to poor classifier performance (see Tables S1 and S2 in the Supporting Information for associated metrics). The subset of *Micromedex* descriptions associated with *BNF* cases that belonged to either of those two advice categories were manually annotated as a surrogate measure of concordance.

The classification of *DIR intersection list* entries in each DIR in terms of clinical management advice is shown in Table S3 in the Supporting Information. In the *BNF*, no advice was available in over half (56.41%) of the DDIs under consideration. The most common advice category was *Avoid* (32.12%) and the least frequently mentioned was *Use alternative* (0.01%). In *Thesaurus*, *Monitor* and *Avoid* jointly covered more than half of the total DDIs (34.89% and 30.82%, respectively), while recommendations related to *Space dosing times*, *Use with caution* and *Wash‐out* were only found in small percentages (1.98%, 1.15% and 0.99%, respectively). In *Micromedex*, the labelling process that was facilitated by sentence classifiers provided the following results: 63.43% of the DDIs were characterised as containing advice related to *Monitor*, 47.02% related to *Avoid* and 35.77% related to *Adjust dose*; low percentages represented *Use alternative* (3.79%), *Discontinue* (2.74%) and *Wash‐out* (0.39%) categories. In 5.38% of the *Micromedex* DDIs, no advice label was assigned.

**FIGURE 4:**
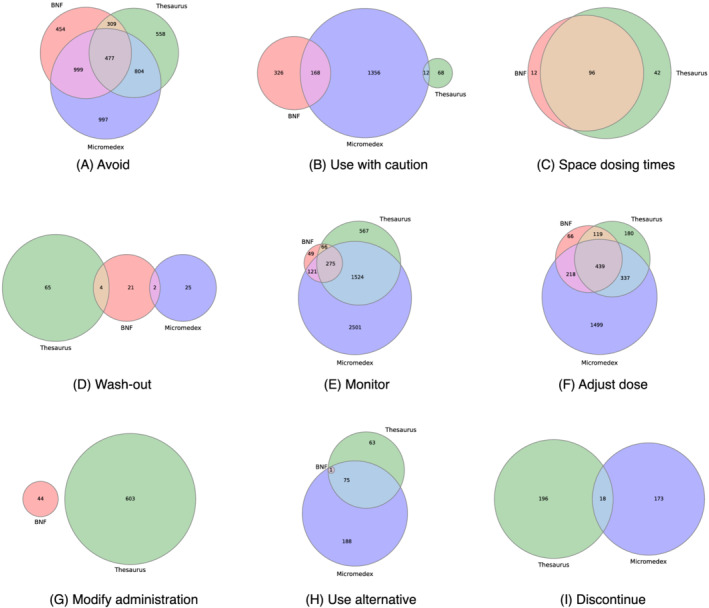
Venn diagrams of the overlap in clinical management advice relating to drug–drug interactions in the drug information resources

The overlap in terms of the DDI‐related advice labels for the DDIs found in the *DIR intersection list* is illustrated using Venn diagrams (Figure 4). The *BNF* and *Thesaurus* did not share any DDIs in their *Modify Administration* and *Use with caution* categories, as opposed to the DDIs found in their *Space dosing times* and *Adjust dose* categories, which showed extensive overlap. *Thesaurus* and *Micromedex* did not have any common DDIs classified into their *Wash‐out* advice categories. Also, for *Wash‐out* and *Use with caution* advice categories, there was little agreement between any two DIRs. The three DIRs overlapped to a high degree in the *Monitor* category. In the majority of *Space dosing times BNF* cases (87.96%), *Micromedex* also contained the respective advice. For *Modify administration*, *Micromedex* included this advice for less than half (43.18%) of the *BNF* cases.

## 4 DISCUSSION

This study reports on the consistency of DDI‐related information included in three major clinical DIRs from different geographic locations, namely the *British National Formulary* (*BNF*), *Thesaurus* and *Micromedex*. The DIRs differed in size and number of ingredients mentioned in the DDI sections. The number of ingredients in *Micromedex* was almost twice that found in the other two DIRs. This is most likely to have been due to the fact that the *BNF* and *Thesaurus* only include medicines licensed in their countries of origin (i.e., UK and France, respectively), while Micromedex includes a broader set of medicines. Although DIR ingredients overlapped to a significant extent, especially between *BNF* and *Thesaurus*, this overlap was not reflected on the DDI sets, which generally showed poor agreement. The *BNF* and *Thesaurus* shared the largest number of DDIs, in contrast to *Thesaurus* and *Micromedex*, which had the fewest DDIs in common.

Our study represents the most comprehensive assessment of the overlap in content and advice provided by different DIRs. However, our findings are consistent with previous studies. For instance, a study that analysed DDIs of fewer than 100 medicines reported less than 7% overall agreement among the examined sources.^8^, ^9^ A more recent analysis that compared three commercial DIRs in terms of listing and severity ranking of DDIs also identified very poor overlap (5%), although DDIs flagged as minor were not considered.^14^

Severity ratings were not consistently reported in the *BNF*, as opposed to *Thesaurus* and *Micromedex*, where ratings were available in all cases. DDIs labelled as critical comprised approximately one fourth of *BNF* and more than 70% of *Micromedex*, in contrast to *Thesaurus*, where the least significant category was the most populous. *Micromedex* and *Thesaurus* showed similarity in their ratings across the different levels of severity. Between *BNF* and *Thesaurus*, their least significant categories (i.e., *Unknown* and *Take into consideration*, respectively) appeared to have a higher level of agreement. However, there was less concordance between *BNF* and *Thesaurus* on the classifications of DDIs in the high severity ratings, with the DDIs classified as *Severe* in the *BNF* being spread across the different *Thesaurus* categories. In terms of *BNF* and *Micromedex*, there was generally better agreement between their critical ratings than with the less severe ones. Apart from the *BNF*‐*Thesaurus* pair, the percentage of DDIs missing from a DIR increased as one moved to DDIs characterised as less severe by another DIR (i.e., increasing trend in the *Not found* column percentages as we go from top to bottom in the tables from Figure 2). *Micromedex* categorised the largest proportion from the *DIR intersection list* as being critical compared to the other DIRs, while around one fourth of the DDIs in the *DIR intersection list* were simultaneously labelled as critical by all three DIRs. Also, the pairwise intersections of DIRs covered larger proportions of the DDIs from the critical severity levels compared to the corresponding proportions from lower severity categories.

Although early studies concluded that significant discrepancies exist in severity ratings between DIRs, Fung et al.’s study advocated the presence of higher levels of agreement than previously reported, especially for the most severe DDIs.^8^, ^9^, ^14^ Our results, suggesting better agreement between critical severity ratings between *BNF* and *Micromedex*, are partially in line with this observation.

Evidence categorisation was not available in *Thesaurus*, thus preventing a comprehensive assessment of the concordance of evidence rating amongst all the DIRs. In the *BNF*, evidence ratings were available for around one third of the DDIs, while they were consistently reported in *Micromedex*. In both DIRs, *Theoretical* was the most frequent category. However, the study revealed a lack of consistency between *BNF* and *Micromedex*, with no cases of strong agreement between any pairs of evidence ratings. An interesting observation was related to the DDIs included in one but missing from the other DIR, as the percentage of *Not found* DDIs in both cases increased as the evidence rating in the other DIR decreased. A study by Vitry that performed a similarity assessment of evidence ratings also highlighting inconsistencies in the grading system for evidence among the different sources.^8^ In terms of clinical management recommendations, there was significant disagreement among the DIRs related to some types of advice, such as *Use with caution*, *Wash‐out*, *Discontinue* and *Modify administration*. Other types (i.e., *Avoid* and *Use alternative*) showed a moderate level of agreement, while *Space dosing time*, *Monitor* and *Adjust dose* demonstrated higher levels of concordance.

### 4.1 Impact on clinical decision support

Examples of missed medicines and DDIs from the different DIRs can be found in Table 5. Some ingredients were surprisingly missing from the DDI section of some of the DIRs, such as levetiracetam from *Thesaurus* and rupatadine from *Micromedex*. A few ingredients were not licensed in the respective countries (e.g., enalaprilat, an intravenous formulation of enalapril, is not available in the UK or France) or had been discontinued (e.g., ketorolac in France or maprotiline in the UK) at the time of data collection. *Micromedex*, however, contained drugs that were discontinued or not approved in the US.

**TABLE 5:** Some examples of ingredients and drug–drug interactions not included in the drug–drug interaction sections of the three drug information resources

Drug information resource	Ingredients	Drug–drug interactions
*BNF*	Aprotinin Dextromethorphan Dibucaine Dimercaprol Filgrastim Flumazenil Goserelin	Methylphenidate – Bupropion Rasagiline – Metoclopramide Bortezomib – Yellow fever vaccine Ephedrine – Midodrine Midostaurin – Lumacaftor Pentamidine – Domperidone Flecainide ‐ Propafenone
*Thesaurus*	Abacavir Beclomethasone Dimercaprol Diphenoxylate Filgrastim Levetiracetam	Ondansetron – Salmeterol Ondansetron – Sunitinib
*Micromedex*	Adefovir Dalfampridine Daratumumab Mifamurtide Rupatadine Zoledronic acid	Ephedrine – Moclobemide Tadalafil – Voriconazole

The difference in size might also be partly related to the nature of the different DIRs. *Micromedex* is a commercial knowledge base, while *BNF* and *Thesaurus* are maintained by professional and regulatory bodies, respectively. Commercial knowledge bases may be overinclusive to minimise potential legal consequences arising from their decision to omit DDIs.

There were a few important DDIs missing from one of the three DIRs (Table 5). Examples include: voriconazole (a CYP3A4 inhibitor) interacting with tadalafil (a PDE‐5 inhibitor) by increasing its systemic exposure^28^; bupropion and methylphenidate, indirect sympathomimetic agents that lower seizure threshold^29^; and sunitinib and ondansetron, which both prolong the QT interval which predisposes to torsade de pointes.^30^

Severity ratings also varied for DDIs in the three DIRs which may impact patient safety. For example, the combination of paroxetine and tramadol was categorised at the lowest severity level (*Take into consideration*) in *Thesaurus* while it was ranked as *Severe* and *Major* in the *BNF* and *Micromedex*, respectively. This is a complex interaction, which leads to decreased plasma concentrations of the active metabolite of tramadol (M1) because of CYP2D6 inhibition by paroxetine, and also an increased risk of serotonin syndrome. Interestingly, the updated version of *Thesaurus* (2020) has upgraded the severity level of the drug pair to *Not recommended*. Other examples include (a) the interaction between cytarabine and flucytosine, which was categorised as *Severe* in the *BNF* (because of decreased concentrations of flucytosine), but at the lowest level in both *Micromedex* and *Thesaurus*; (b) the interaction between niacin and statins, which increases the risk of myopathy and rhabdomyolysis, was characterised as *Severe* in the *BNF* and *Major* in *Micromedex* but was completely missing in *Thesaurus*; and (c) the combination of non‐steroid anti‐inflammatory drugs with thiazide‐type diuretics (e.g., chlorothiazide, chlortalidone) was ranked as *Severe* in the *BNF* and *Major* in *Micromedex* (but as *Precautions for use* in *Thesaurus*) because of the risk of acute renal failure.

### 4.2 Strengths and limitations

As opposed to multiple previous efforts to assess the level of agreement among DIRs in terms of DDI information, this study examined entire resources, thus revealing the relative size of information in each of the DIRs and exploring the stratification of the included DDIs in terms of severity, evidence and clinical management recommendations. To the best of our knowledge, this is the first comprehensive effort to compare clinical advice for managing DDIs that is provided in multiple DIRs, with a clear focus on clinically oriented sources compared to previous work.^10^ While a previous study expanded the comparison of DIRs at multiple levels (i.e., clinical drug, ingredient and drug class),^14^ our analysis was limited to the ingredient level. This standardisation of DIRs enabled a “fair” comparison in terms of the volume of information listed. Also, code availability for data extraction and standardisation will enable reproducibility of the analysis.

However, there are limitations to this study. First, no updates have been taken into consideration since the date of data retrieval (i.e., offline data). Therefore, the results and conclusions of this study provide an overview of their similarity and consistency at that specific point in time, although no major updates usually occur. Second, *Thesaurus* contained a few DDIs originally reported at the drug class level, which were associated with multiple severity ratings. Hence, some standardised DDIs at the ingredient level were assigned more than one severity rating. In the *BNF*, there was a limited number of DDIs having multiple severity levels depending on the described clinical outcome. In both cases, the highest severity rating was considered for further analysis. Another limitation could be related to the different countries of origin for the DIRs that were considered in this study, which might have contributed to a small extent to the discrepancies observed. Other limitations include the comparison of clinical management options only for the DDIs present in the intersection of the DIRs and the custom‐made labelling process applied to *Micromedex*. Additionally, in the *BNF*, there were referrals to the guidance section of the website for various drug categories that were left unmapped during the annotation process, e.g., “*For FSRH guidance, see contraceptives, interactions*”, or “*See ‘serotonin syndrome’ and ‘monoamine‐oxidase inhibitor’ under antidepressant drugs for more information and for specific advice on avoiding monoamine‐oxidase inhibitors during and after administration of other serotonergic drugs*”. In this way, the overall advice support provided by the *BNF* might have been underestimated, although no concrete clinical advice was provided.

For future work, it would be interesting to evaluate a larger number of DIRs and include DIRs (e.g., Medscape, Lexicomp, Stockley's Drug Interactions) which could not be accessed in this instance due to lack of a subscription or due to terms and conditions that currently prohibit the type of analysis we have conducted. It would also be interesting to explore the completeness of generic DIRs as opposed to resources tailored to specific drug categories (e.g., the Liverpool Drug Interaction Checkers for anticancer drugs, etc.) that would be expected to provide more complete information. The evaluation of agreement on various types of DDI‐related information among DIRs from the same country of origin would be another relevant topic for future research, although significant levels of discordance would not be surprising, similar to previous studies.^14^ Additionally, more comprehensive efforts to compare clinical management recommendations among entire resources would be beneficial from a clinical perspective.

### 4.3 Implications and conclusions

It is reasonable to assume that the inclusion of clinically significant DDIs in DIRs would improve drug efficacy and reduce adverse reactions. There is, however, a balance to strike since the value of these tools could be diminished if too many minor or clinically insignificant DDIs are included in an effort to limit legal liability.^31^ This leads to the phenomenon of alert fatigue where practitioners ignore the alerts provided by the system due to the sheer volume of generated alerts,^32^ with important clinical consequences for patient safety.

The Evidence workgroup from the DDI CDS Conference Series has highlighted the importance and need for higher‐quality information related to DDIs and also suggested the establishment of systematic DDI search criteria in order to determine the existing evidence related to the information provided.^33^ Our analysis also shows the need for consistency in the definitions of severity and evidence ratings provided by the various DIRs. The availability of DDI evidence in a standardised format with adequate literature support, where possible, can improve prescribing decisions by allowing the prescriber to refer to appropriate resources and use clinical judgement in case of doubt.

The inclusion of clinical management options for DDIs in CDS tools is also quite important and especially useful in the clinical setting, as there is no single response to a potential DDI. More focus on this aspect has been suggested in multiple studies, which advocate more detailed and actionable advice (i.e., what and when to monitor) and clear indications of the strength of the recommendation.^34^, ^35^ We recommend that, by providing a dedicated section for clinical management recommendations that contains clear, actionable recommendations, information retrieval in the clinic can be facilitated, and potentially improve individualised risk–benefit assessment of a specific DDI. In cases where the benefits of a drug combination outweigh its risks, strategies to mitigate potential adverse outcomes (e.g., therapeutic drug monitoring, vital signs, discontinuation of one of the drugs, etc.) should be provided and will improve the benefit–harm balance of the drug combination. In the future, information about specific patient risk factors for DDIs, such as genetic polymorphisms, could also be included to enhance DDI preventability.

The use of pharmacologic drug classes in *Thesaurus* to summarise DDIs might become a source of confusion for clinicians. In many cases, individual drugs from a drug class have different pharmacological profiles (e.g., excretion, metabolism, etc.), which contribute to a markedly different DDI risk when considering the same interacting drug.^34^ Consequently, drug ingredient indexing may paradoxically impede searching rather than achieve the aim of providing effective DDI summaries.

In conclusion, there is a great deal of interest in clinical decision support systems providing information on DDIs to optimise medicines use so that the use of drug combinations that affect either efficacy and/or safety can be avoided. However, there is a lack of consistency and standardisation in the information provided by different DIRs. Our study, which has systematically compared three DIRs, shows that there is considerable variation in the DDI information provided in these resources. Such variability in information could have deleterious consequences for patient safety, and there is a need for harmonisation and standardisation.

## COMPETING INTERESTS

E.K. receives a PhD studentship that is jointly funded by AstraZeneca and the EPSRC. She also worked on a fixed‐term employment contract for AstraZeneca when this article was prepared. A.B. is an employee of Ceva Santé Animale. M.P. has received partnership funding for the following: MRC Clinical Pharmacology Training Scheme (co‐funded by MRC and Roche, UCB, Eli Lilly and Novartis); and grant funding from Vistagen Therapeutics. He also has unrestricted educational grant support for the UK Pharmacogenetics and Stratified Medicine Network from Bristol‐Myers Squibb and UCB. He has developed an HLA genotyping panel with MC Diagnostics, but does not benefit financially from this. M.P. is part of the IMI Consortium ARDAT (www.ardat.org). B.D. was AstraZeneca's employee and shareholder when this article was prepared but has since ended his relationship with both. S.M. has no conflict of interest to declare.

## CONTRIBUTORS

M.P., S.M. and E.K. conceived the work and designed the analysis. M.P., S.M., E.K. and B.D. contributed to data interpretation. E.K. conducted the data extraction, performed the analysis and drafted the original manuscript. A.B. contributed to data analysis. M.P., S.M. and B.D. provided critical feedback. All authors contributed to revising the paper and approved the final manuscript.

## Supporting information


**Figure S1.** An overview of drug–drug interaction online resources
**Figure S2.** Pipeline for clinical recommendation labelling
**Figure S3.** Similarity matrix of the Jaccard index for all drug information resource severity ratings
**Table S1.** Performance metrics and applied thresholds of the selected sentence classifiers for *Micromedex* descriptions.
**Table S2.** Evaluation of selected classifiers using an independent validation subset in terms of positive predictive value (PPV), sensitivity and F1‐score metrics (%).
**Table S3.** Number and percentage of drug–drug interactions included in the *DIR intersection list* by advice label for each drug information resource.

## Data Availability

Data from the DIRs were derived from the following web sources: the BNF website (https://bnf.nice.org.uk/interaction/, available in the public domain); ANSM website (https://ansm.sante.fr/documents/reference/thesaurus-des-interactions-medicamenteuses-1, available in the public domain). Restrictions apply to the availability of Micromedex data, which were used under license for this study. Data are available at https://www.micromedexsolutions.com/ with the permission of IBM Watson Health. The code that supports the web data extraction and analysis are available at: https://github.com/elpidakon/CRESCENDDI.
