# Supplementary material for: Similarity and consistency assessment of three major online drug–drug interaction resources
Source: Br J Clin Pharmacol. 2022 Apr 12;88(9):4067–79. doi: 10.1111/bcp.15341 (PMC9545693; doi:10.1111/bcp.15341)
Supplement: Supplementary file 1 — Figure S1. An overview of drug–drug interaction online resources Figure S2. Pipeline for clinical recommendation labelling Figure S3. Similarity matrix of the Jaccard index for all drug information resource severity ratings Table S1. Performance metrics and applied thresholds of the selected sentence classifiers for Micromedex descriptions. Table S2. Evaluation of selected classifiers using an independent validation subset in terms of positive predictive value (PPV), sensitivity and F1‐score metrics (%). Table S3. Number and percentage of drug–drug interactions included in the DIR intersection list by advice label for each drug information resource. [file BCP-88-4067-s001.docx]

**SUPPLEMENTARY INFORMATION**

**Figure S1.** An overview of drug-drug interaction online resources.


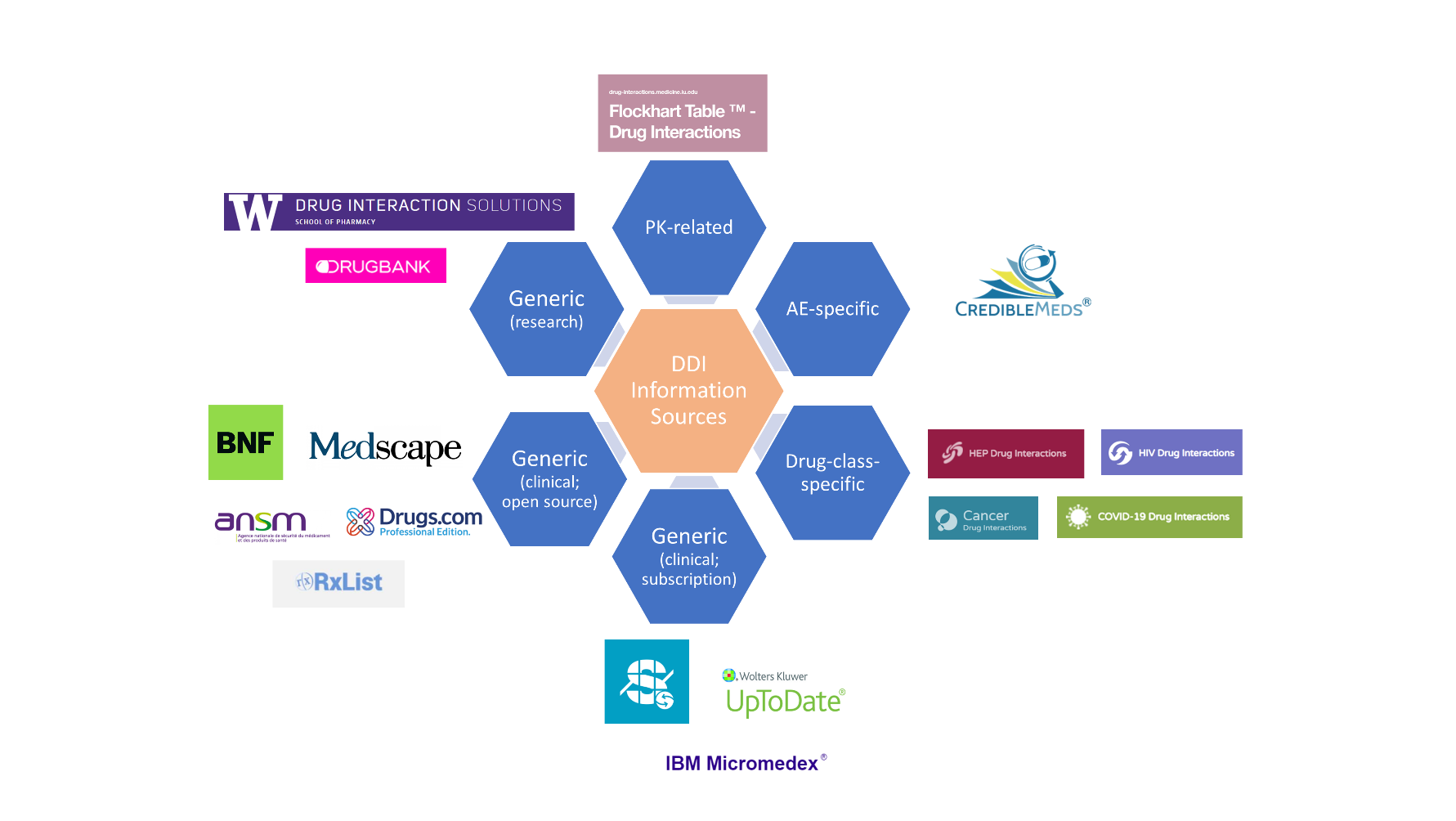


**Figure S2.** Pipeline for clinical recommendation labelling.


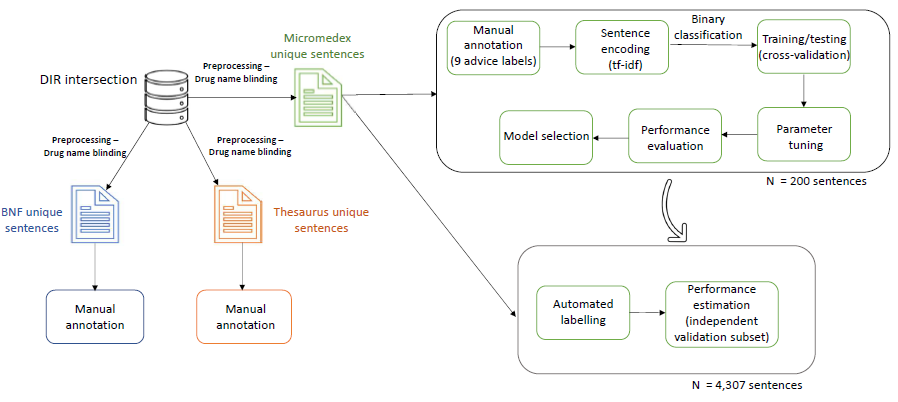


**Figure S3.** Similarity matrix of the Jaccard index for all drug information resource severity ratings.


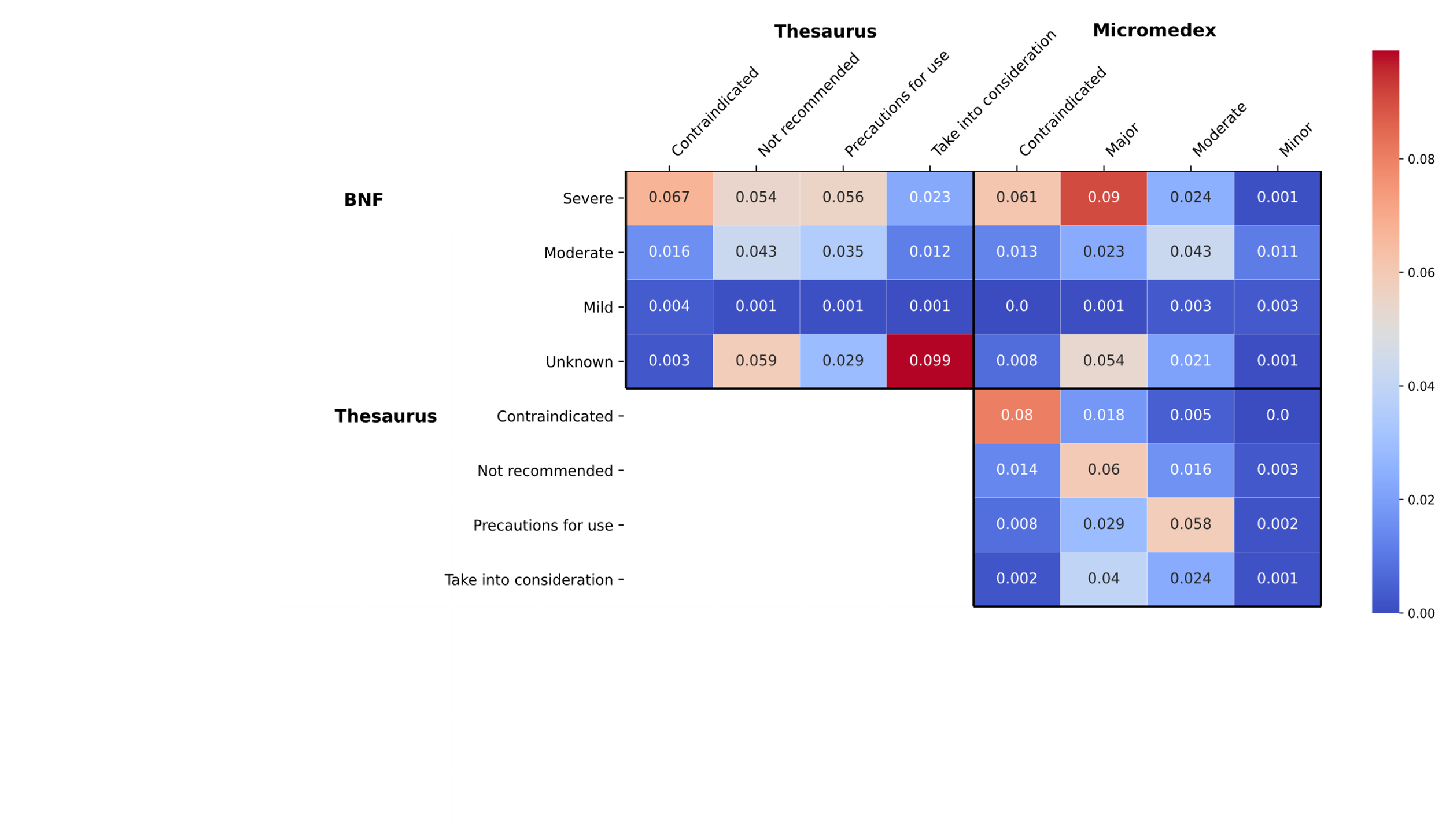


**Table S1:** Performance metrics and applied thresholds of the selected sentence classifiers for *Micromedex* descriptions.

| **Advice label** | **PPV** | **sensitivity** | **threshold** |
| --- | --- | --- | --- |
| **ADJUST DOSE** | 0.860000 | 1.000000 | 0.352397 |
| **AVOID** | 0.804878 | 0.750000 | 0.459679 |
| **DISCONTINUE** | 1.000000 | 0.500000 | 0.653243 |
| **MONITOR** | 0.892308 | 1.000000 | 0.356476 |
| **USE ALTERNATIVE** | 0.833333 | 0.714286 | 0.367563 |
| **USE WITH CAUTION** | 0.800000 | 0.923077 | 0.210383 |
| **WASH-OUT** | 1.000000 | 0.142857 | 0.858192 |

**Table S2.** Evaluation of selected classifiers using an independent validation subset in terms of positive predictive value (PPV), sensitivity, and F1-score metrics (%).

| **Classifier** | **PPV** | **sensitivity** | **F1-score** |
| --- | --- | --- | --- |
| **AVOID** | 78.26 | 85.71 | 81.82 |
| **USE WITH CAUTION** | 100.00 | 85.71 | 92.31 |
| **MONITOR** | 82.76 | 100.00 | 90.57 |
| **ADJUST DOSE** | 90.32 | 100.00 | 94.92 |
| **USE ALTERNATIVE** | 71.43 | 71.43 | 71.43 |
| **WASH-OUT** | N/A | 0 | N/A |
| **DISCONTINUE** | N/A | 0 | N/A |

**Table S3.** Number and percentage of drug-drug interactions included in the *DIR intersection list* by advice label for each drug information resource.

| **Advice label** | **BNF** | **Thesaurus** | **Micromedex** |
| --- | --- | --- | --- |
| **AVOID** | 2,239 (32.12%) | 2,148 (30.82%) | 3,277 (47.02%) |
| **USE WITH CAUTION** | 494 (7.09%) | 80 (1.15%) | 1,536 (22.04%) |
| **MONITOR** | 511 (7.33%) | 2,432 (34.89%) | 4,421 (63.43%) |
| **ADJUST DOSE** | 842 (12.08%) | 1,075 (15.42%) | 2,493 (35.77%) |
| **WASH-OUT** | 27 (0.39%) | 69 (0.99%) | 27 (0.39%) |
| **SPACE DOSING TIMES** | 108 (1.55%) | 138 (1.98%) | 95/108^[[1]](#footnote-1)^ (87.96%) |
| **MODIFY ADMINISTRATION** | 44 (0.63%) | 603 (8.65%) | 19/44^[[2]](#footnote-2)^ (43.18%) |
| **DISCONTINUE** | N/A | 214 (3.07%) | 191 (2.74%) |
| **USE ALTERNATIVE** | 1 (0.01%) | 139 (1.99%) | 264 (3.79%) |
| **NOT MENTIONED** | 3,932 (56.41%) | 2,793 (40.07%) | 375 (5.38%) |

1. BNF cases for *Space dosing times* [↑](#footnote-ref-1)
2. BNF cases for *Modify administration* [↑](#footnote-ref-2)
